# Impact of Pesticide Contamination on Aquatic Microorganism Populations in the Littoral Zone

**DOI:** 10.1007/s00244-012-9852-6

**Published:** 2012-12-11

**Authors:** S. Lew, M. Lew, A. Biedunkiewicz, J. Szarek

**Affiliations:** 1 Faculty of Biology and Biotechnology, University of Warmia and Mazury in Olsztyn, Oczapowskiego 1A, Olsztyn, Poland; 2 Faculty of Veterinary Medicine, University of Warmia and Mazury in Olsztyn, Oczapowskiego 2, Olsztyn, Poland

## Abstract

The effect of pesticide contamination of the littoral zone on the population of bacteria and fungi was analyzed using the example of a eutrophic water reservoir exposed for >30 years to the influence of expired crop-protection chemicals, mainly DDT. For three consecutive years, quantity analyses of bacteria and fungi were conducted and the composition of the microorganism population analyzed against seasonal dynamics. Mold and yeast-like fungi were also isolated and identified. Within the *Bacteria* domain, in addition to the large groups of microorganisms (Alphaprotobacteria, Betaprobacteria, and Gammaproteobacteria, Actinobacteria, and Cytophaga-Flavobacterium), the analysis also involved the presence of bacteria predisposed to degraded pesticides in natural environments: *Pseudomonas* spp. and *Alcaligenes* spp. The quantity dynamics of aquatic microorganisms indicated that bacteria and fungi under the influence of long-term exposure to DDT can adapt to the presence of this pesticide in water. No modifying effect of DDT was observed on the quantity of microorganisms or the pattern of seasonal relationships in the eutrophic lake. Changes were shown in the percentage share of large groups of bacteria in the community of microorganisms as was an effect of contamination on the species diversity of fungi. The data show the effectiveness of aquatic microorganism–community analyses as a tool for indicating changes in the water environment caused by pesticide contamination.

Pesticide presence in natural ecosystems results from wide application in contemporary agriculture systems, application over many years, and the storage of pesticides past their expiration date in disposal sites unsuitable for this purpose (Carabias-Martinez et al. 2003; Cerejeira et al. 2003; Konstantinou et al. 2006; Hildenbrandt et al. 2008; Foit et al. 2010). Some of these preparations, currently withdrawn from use, are listed as persistent organic pollutants (POPs). The danger they pose results both from their acute toxicity and durability in the natural environment (Benimeli et al. 2003).

In ecosystems with an established presence of hazardous substances of anthropogenic origin, multidirectional and long-term (related to accumulation phenomenon) effects of pesticides have been observed (Fleeger et al. 2003; Manadori et al. 2006; Singh 2009; Skibniewska 2010). They affect organisms living in the natural environment, including those that are not their immediate target, which can consequently lead to changes in the quality and quantity composition of a population. Natural populations exhibit a number of responses to these contaminants, and the quickest reaction has been observed from microorganisms at the base of the trophic cascade: bacteria and fungi (DeLorenzo et al. 2001; Lew et al. 2011; Foit et al. 2010). These organisms can make use of substances entering the environment as feeding substrate for a source of energy or a building material, thus affecting balance in the ecosystem (Buesing and Gesser 2006). They are highly efficient in transforming organic pollutants entering their environment. They modify the structure of these compounds and their toxic properties, in many cases leading to complete mineralization of organic components into inorganic products (Zipper et al. 1996; Kalwasińska et al. 2010).

The littoral zone is a transit zone between the terrestrial and aquatic environment. It is a biodiversity hot spot and home to many aquatic and terrestrial bacteria and fungi as well as a place where many energy-transformation processes take place (Wu et al. 2010). With macrophytes covering it and plants growing in this area, as well as microorganisms dwelling in open waters, this zone acts as a kind of filter protecting the ecosystem against harmful substances (Glinska-Lewczuk et al. 2009; Obolewski et al. 2009). A high concentration of organic substances and generally good thermal-aerobic conditions make this zone a place where autotrophic and heterotrophic bacteria and fungi can flourish (Kalwasińska et al. 2010). As a result of interest in this zone and its communities, many studies have focused on evaluating microbiological responses to exposure to various pesticides (DeLorenzo et al. 2001; Fleeger et al. 2003; Foit et al. 2010). However, the number of publications describing the reactions of bacteria and fungi to these substances in natural ecosystems is limited (Pesce et al. 2008; Leboulanger et al. 2011; Lew et al. 2011). This scarcity of studies has led us to pursue further research to understand how microorganisms in water ecosystems react to these POP exposures.

The subject of present work, therefore, was assessing the effect of chloro-organic pesticide DDT—a pesticide that has generally been withdrawn from use but persists in the environment—on the composition quality and quantity of bacteria and fungi in the water ecosystem. The aim of the research was to establish effects after many years of surface runoff into the lake from a nearby hazardous substance disposal site on the community of bacteria and fungi in the littoral zone of the water reservoir.

## Materials and Methods

### Study Site and Sampling

Sites located in the littoral zones of lakes Szeląg Wielki (Lit-1 exposed site and Lit-2 unexposed sites) and Ruda Woda (Lit-control) were selected for the research. These reservoirs are characterized by similar morphometric parameters (Table 1) in being eutrophic-type finger lakes. The lakes feature a poorly developed coastline with narrow littoral zones that do not exceed 15 % of the lake area. Phytolittoral is composed here of plants of the Potamogetonetea class and the class of Phragmitetea, which is a characteristic community for eutrophic lakes (Grzybowski et al. 2005). They are situated in northeastern Poland, surrounded by forest complexes, and separated by a distance of 50 km. A reclaimed area after pesticide-tomb liquidation in 2004 is situated in the direct drainage area of Lake Szeląg Wielki in the vicinity of the littoral zone (Lit-1 exposed site) on a sandy hill (104 m above sea level). This disposal site of plant-protection chemicals past their expiration date (mainly DDT) existed for 30 years and could hold 78 Mg pesticides; however, at the moment of extraction, it contained 54 Mg pesticides. From here, as a result of washing and erosion of soil from the area left after the pesticide dump, pesticides reached the water reservoir with rainfall as well as with a small stream flowing through this area and carrying water to Lake Szeląg Wielki, thus affecting the organisms inhabiting the lake, including bacteria and fungi (Grzybowski et al. 2005; Lew et al. 2011). The Lit-2 unexposed site is located in the opposite direction of the Lit-1 exposed site. No pesticide dump or area reclaimed after a pesticide-tomb release has been located in the vicinity of Lake Ruda Woda, and the littoral site (Lit-control) at this reservoir provided a control sample for our studies.

**Table 1:** Morphometric data of the water of lakes Szeląg Wielki and Ruda Woda

Morphometric date/lake	Szeląg Wielki	Ruda Woda
Area (ha)	599.0	654.1
Volume × 10^3^ (m^3^)	81,111.2	69,324.0
Maximal depth (m)	35.5	27.8
Mean depth (m)	13.5	10.6
Maximal length (m)	12,500	12,050
Maximal width (m)	900	1 750

Water samples from the littoral zones of the lakes were collected from spring 2008 to autumn 2010 at 2 month intervals. Samples were collected from the subsurface layer –0.15 to 0.25 m from two sites on Lake Szeląg Wielki ([1] the littoral zone reached by the stream flowing from the reclaimed area [Lit-1 exposed site] and [2] the littoral zone on the opposite side of the lake [Lit-2 unexposed site]) as well as one littoral site on Lake Ruda Woda (Lit-control).

### Hydrochemical Parameters

Selected hydrochemical parameters were measured along with a collection of microbiological samples. A multiparameter water-quality meter YSI 6600 (Xylem Inc., Yellow Springs, USA) was used to determine temperature (T  °C), dissolved oxygen (DO μg_*_l^−1^), and pH. The content of DDT and its metabolites (DDE, DDD, and DDT) was determined with the method proposed by Amarowicz et al. (1986) using a PU 4600 gas chromatograph and by Ludwicki et al. (1996) in a modified version adapted for water analyses. The accuracy of measurements of chloro-organic insecticides in water was calculated with the formula W = [(a-b)/c] × 100, where W denotes the recovery coefficient expressed as a percentage value, a indicates the amount of standard determined in an enriched sample, b stands for the amount of standard determined in a blind sample, whereas c denotes the added amount.

### Total Bacterial Abundance

Bacterial abundance was determined by epifluorescence microscopy (Porter and Feig 1980). Triplicate subsamples were fixed with neutralized formaldehyde (pH 7.4; final concentration 4 %) and stained with DAPI (Sigma-Aldrich, St. Louis, MO, USA) final concentration 0.01 μg mL^−1^ for 15 min in the dark. The samples were then filtered gently through 0.2-μm black Nuclepore filters (type GTTP; Millipore). Bacteria were counted under an Olympus epifluorescence microscope. More than 1000 bacterial cells in 20 objective fields were counted.

### Total Fungal Abundance

To determine the number of fungi colonies, the method of membrane filters was used (Qureshi and Dutka 1976; Biedunkiewicz and Baranowska 2011). A total of 500 mL water was filtered through a membrane filter (FMW-5/50), placed on dishes with solid Sabouraud’s medium, and incubated for 48 to 72 h at 37 °C. Afterward, yeast fungi colonies were counted, sieved onto Sabouraud’s agar slant with chloramphenicol, and then incubated again at 37 °C for 48 to 72 h. The analysis was performed in three replications for each site and for all collection points of littoral water samples (Biedunkiewicz-Ziomek and Dynowska 2004).

### Microorganisms of Littoral Zone Composition

#### Bacteria

Samples for community analysis were fixed in freshly buffered prepared paraformaldehyde (pH 7.4) to a final concentration of 2 % (vol/vol) and stored for several hours at 4 °C. The samples were filtered through white polycarbonate filters (type GTTP; Millipore), rinsed twice with sterile water, dried at room temperature, and stored at –20 °C. Bacterial community composition was investigated by fluorescent in situ hybridization (FISH) with the use of Cy3-labeled oligonucleotide probes in accordance with the hybridization procedure for aquatic microorganisms proposed by Pernthaler et al. (2001a, b). The rRNA-targeted oligonucleotide probes were used to detect bacteria of the ALF 968, -BET42a, and GAM42a subdivisions of Proteobacteria and of the Cytophaga-Flavobacterium cluster CF319a (Manz et al. 1992). Gram-positive bacteria with a high concentration of GC-Actinobacteria were identified using an HGC 69a probe (Roller et al. 1994). To determine the proper bacteria belonging to the Eubacteria group, an EUB 338_I-III_ probe was used (Daims et al. 1999). To detect microorganisms capable of decomposing pesticides (mainly DDT), a PAE 997 probe was chosen for *Pseudomonas* spp. and an ALBO 557 for *Alcaligenes* spp. (Friberg-Jansen et al. 2003).

Bacterial cells on the filter sections were observed with an epifluorescent microscope equipped with filter sets for DAPI and Cy3. The fractions of FISH-stained bacteria in at least 1000 DAPI-stained cells/sample were quantified.

#### Fungi

Diagnostics for yeast-like fungi were performed on the basis of morphological and biochemical features (zymograms and auxanograms). The macroscopic assessment was performed on the basis of features of the colonies developed, and microscopic characteristics of fungi were examined in in vivo preparations in a drop of water dyed with methylene blue and in microcultures incubated for 48, 72, and 144 h (Biedunkiewicz-Ziomek and Dynowska 2004) at 37 °C on Nickerson substrate. Colonies of mold fungi from the Sabouraud’s substratum were sieved onto dishes with a Czapek-Dox substrate. Preparations were produced from the fungi obtained, applying the imprinting method with the use of adhesive tape, and dyed with methylene blue with lactophenol according to Gerlach (1972). Mold fungi were identified on the basis of the morphology and macroscopic features of the mycelium formed (De Hoog et al. 2000; Howard 2003; Kurtzmann and Fell 2000; Lodder and Kreger-van Rij 1967; Midgley et al. 1997; Raper and Fennel 1965; Raper et al. 1949).

### Data Analyses

Samples from the lakes were taken in triplicate to determine the variability of DAPI counts and the count of fungi isolated from the littoral water. Probe-specific cell counts are presented as a percentage of cells visualized by DAPI, and the mean abundances and SDs were calculated using Statistica v.9 software (Statistic Graphic 2010). The impact of hydrochemical parameters on the studied microbiological parameters in the analyzed water samples was determined with canonical correspondence analysis (CCA). The ordination was performed using CANOCO 4.5 Wageningen UR, Netherlands (ter Braak and Šmilauer 2002).

## Results

### Hydrochemical Analyses

**Fig. 1:**
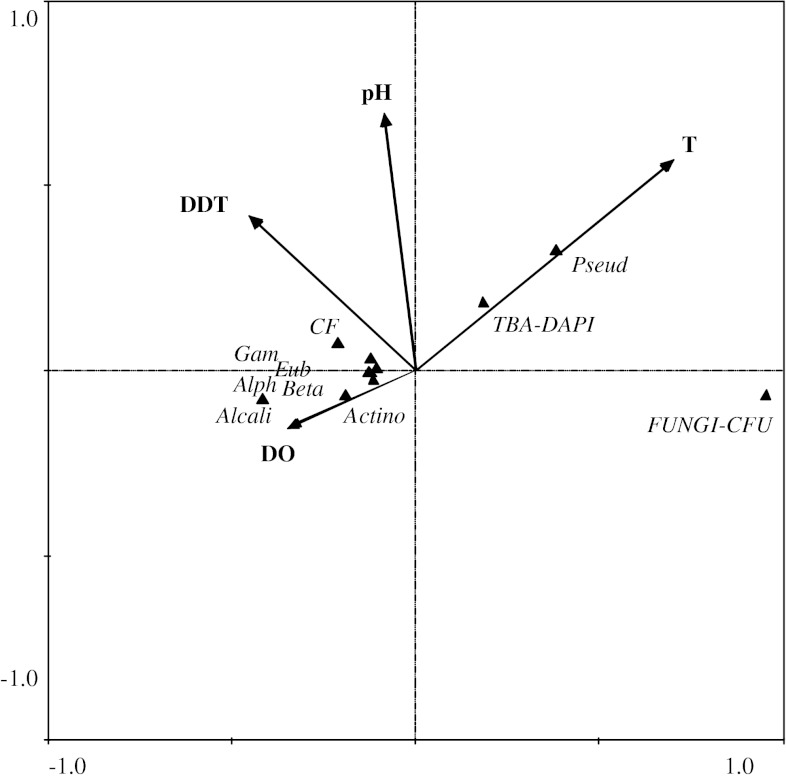
Results of CCA between hydrochemical variables (pH, DDT, temperature [T], dissolved oxygen [DO]) and microbiological parameters in littoral zones. FUNGI-CFU = total number of fungi abundances; *Eub* Eubacteria, *Alph* Alphaproteobacteria, *Beta* Betaproteobacteria, *Gam* Gammaproteobacteria, *Actino* Actinobacteria, *CF* Cytophaga-Flavobacterium, Alcali *Alcaligenes spp*., Pseud *Pseudomonas* spp

Table 2 lists the results of hydrochemical analyses. Water from the littoral zones in all research seasons was characterized by good oxygenation and quite high average annual temperature, which did not decrease below 8 °C. The content of DDT (mg L^−1^) in water was analyzed for all sites. Its presence was observed only at the Lit-1 exposed site. The quantity of DDT detected tended to decrease from early spring to autumn throughout the entire 3-year period of research. Table 3 lists the mean values of this parameter. The hydrochemical variables used in the ordination explain 69.8 % of the total variation of microbiological parameters. Reaction (pH) and temperature were statistically important factors that determined the variation of microbiological parameters (Fig. 1).

**Table 2:** Mean values of selected physicochemical water parameters (T, pH, DO) and the share of DDT (Σ DDT) and its metabolites DDE, DDD, and DDT in water of selected sites in 2008–2010

Parameter season	Szeląg Wielki Lake								Ruda Woda Lake			
	Lit-1 exposed site				Lit-2 unexposed site				Lit-control			
	T (^o^C)	DO (ugl^−1^)	pH	Σ DDT (mgl^−1^)	T (^o^C)	DO (ugl^−1^)	pH	Σ DDT (mgl^−1^)	T (^o^C)	DO (ugl^−1^)	pH	Σ DDT (mgl^−1^)
Early spring	8.4	9.6	7.9	194	8.3	10.7	7.8	0	8.5	9.5	7.9	0
Late spring	15.2	13.0	8.1	175	14.4	12.6	8.5	0	16.5	11.9	8.5	0
Summer	23.9	10.9	8.5	125	23.9	10.6	8.2	0	26.3	8.5	8.3	0
Autumn	17.0	9.9	8.5	26	18.0	9.9	8.0	0	19.0	8.9	8.2	0

*T* temperature, *DO* dissolved oxygen

**Table 3:** Species composition of fungi isolated from the water of lakes Szeląg Wielki and Ruda Woda

Season	Szeląg Wielki		Ruda Woda
	Lit-1 exposed site	Lit-2 unexposed site	Lit-control
Early spring	*A. fumigatus*	*A. fumigatus*	*A. fumigatus*
			*Trichoderma citrinoviridae*
Late Spring	*A. fumigatus*	*A. fumigatus* *Lipomyces*	*A. fumigatus* *T. citrinoviridae*
Summer	*A. fumigatus*	*A. fumigatus*	*A. fumigatus*
		*Exophiala dermatitidis*	
			*A. viridis*
		*Pichia anomala*	
			*Candida glabrata*
			*Debaryomyces vannrijae*
			*E. spinifera*
			*Oosporidium margaritiferum*
			*T. citrinoviridae*
			*T. viride*
Autumn	*A. fumigatus*	*P. guilliermondii*	*Kluyveromyces marxianus*
		*(C. guilliermondii)* *Rhizopus sp.*	
			*K. polysporum*
			*Rhodotorula glutinis, Exophiala spinifera,*
			*T. citrinoviridae*
			*Trichosporon inkin*

### Total Bacterial and Fungal Abundance

**Fig. 2:**
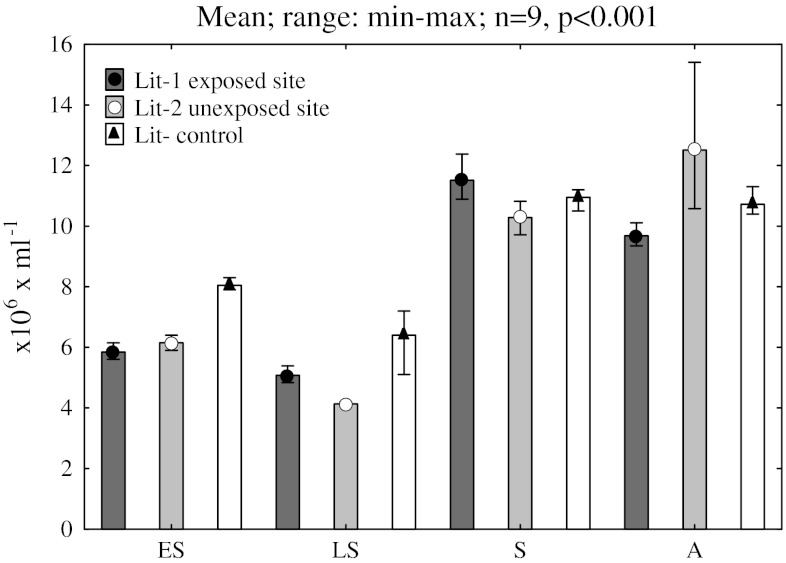
Seasonal change (*ES* early spring, *LS* late spring, *S* summer, *A* autumn) of total number of bacteria abundances (TBA-DAPI) in littoral zones of Lake Szeląg Wielki (*black circle* = Lit-1 exposed site; *white circle* = Lit-2 unexposed site) and Lake Ruda Woda (*black triangle* = Lit-control)

Seasonal analysis concerning the abundance of bacteria from littoral zones of lakes proved that, on average, 10 × 10^6^ mL^−1^ microorganisms were observed at the turn of summer and autumn (Fig. 2). In that period, only slight differences were observed for individual sites. The highest value for a total bacteria count in the littoral zone of Lake Szeląg Wielki, reached by the small water course flowing from the area reclaimed after the pesticide dump (Lit-1 exposed site), was observed in summer; however, almost 2 months later the highest bacterial abundance was found on the opposite shore of the lake (Lit-2 unexposed site). The average bacterial count in spring for all sites did not exceed 8 × 10^6^ mL^−1^.

**Fig. 3:**
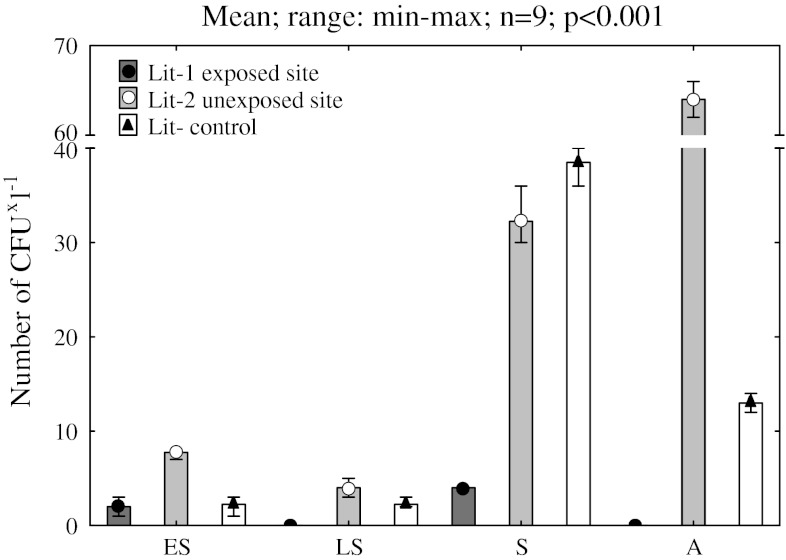
Seasonal change change (*ES* early spring, *LS* late spring, *S* summer, *A* autumn) of total number of fungi abundances (CFU) in the littoral zones of Lake Szeląg Wielki (*black circle* = Lit-1 exposed site; white circle = Lit-2 unexposed site) and Lake Ruda Woda (*black triangle* = Lit-control)

A tendency for seasonal changes in the total count of fungi was also found. Fungi were most abundant in the summer and autumn (Fig. 3). The exception was the Lit-1 exposed site, where fungi were found only in early spring and summer. On the other side of Lake Szeląg Wielki (Lit-2 unexposed site), fungi were isolated from water samples for the entire research period, with highest number in autumn (66 colony-forming units [CFU] mL^−1^).

### Bacterial and Fungal Composition in the Littoral Zone

**Fig. 4:**
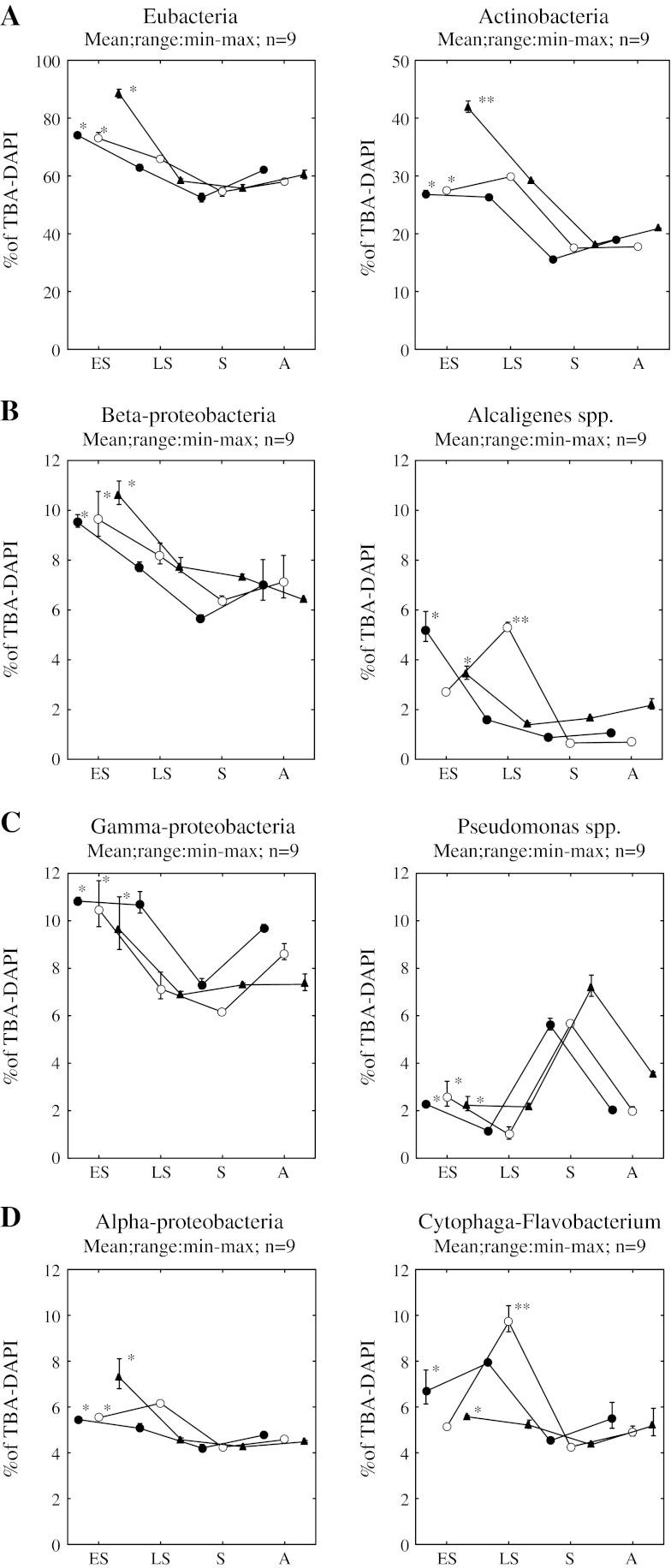
**a**–**d** Seasonal change change (*ES* early spring, *LS* late spring, *S* summer, *A* autumn) of taxonomic composition of the bacterial communities in the littoral zones of Lake Szeląg Wielki (*black circle* = Lit-1 exposed site; white circle = Lit-2 unexposed site) and Lake Ruda Woda (*black triangle* = Lit-control). Values are expressed as the percentage of hybridized cell counts of TBA of DAPI-stained cells. *p < 0.001; **p < 0.05

Eubacteria identified in the water of the littoral zone accounted for 51 % to 90 % of total bacterial abundance (TBA) detected by DAPI (TBA-DAPI) staining (Fig. 4a). For the great part of the research period, >65 % Eubacteria were identified. The group of microorganisms with the most abundant representation was Actinobacteria in the control lake; their share decreased from early spring to summer (40 % on average in relation to TBA-DAPI) to reach a value that was almost half as high in autumn (20 % on average). In Lake Szeląg Wielki, the same pattern of seasonal changes was observed in two littoral sites, with a slight predominance in the Lit-2 unexposed site (Fig. 4a). A similar tendency was found for Betaproteobacteria, which formed the second-largest group of microorganisms in terms of abundance determined in the littoral zones of the examined lakes (Fig. 4b). Within this group, the share of bacteria of *Alcaligenes* spp. was analyzed. Seasonal dynamics results for bacteria identified with the ALBO 557 probe in relation to TBA-DAPI are presented in Fig. 4b. Bacteria of the *Alcaligenes* genus in the control lake formed an even share (20 % to 30 %) within Betaproteobacteria for the entire period of research. In Lake Szeląg Wielki, their share in the spring period was greater, amounting to 30 % in the Lit-1 exposed site and 46 % in the Lit-2 unexposed site regarding all Betaproteobacteria. During the second half of the year, the bacterial abundance, as detected by BET42a probe, significantly decreased with, on average, 15 % and 10 % in the Lit-1 and Lit2 exposed sites, respectively.

Gammaproteobacteria were represented in the community of microorganisms in large quantities. At the Lit-1 exposed site, they were more abundant for the entire research period than at the Lit-2 unexposed and the Lit-control sites (Fig. 4c). Regarding Gammaproteobacteria, particular attention was given to the *Pseudomonas* genus, which was most abundantly recorded in summer (5.5 % on average for Lake Szeląg Wielki and 6.5 % for Lake Ruda Woda relative to all TBA-DAPI bacteria) despite the fact that the count of Gammaproteobacteria in that season was the lowest for the entire period of research.

The two other groups of microorganisms of the Eubacteria type (Alphaproteobacteria and Cytophaga-Flavobacterium) were recorded in the lowest quantities, whereas the pattern of seasonal changes showed a similarity between sites situated on Lake Szeląg Wielki and a slight departure for the control site (Fig. 4d). However, both groups were most abundantly represented during the entire season in late spring, and at all sites their count was the lowest in the summer.

Fungi were regularly observed for the entire research period at the Lit-2 unexposed and Lit-control sites. At the Lit-1 site, where the watercourse carried rainwater from the area reclaimed after the pesticide tomb, fungi appeared in scarce quantities both in very early spring and in the summer. However, at the Lit-2 unexposed site on the same lake, not only were fungi recorded in all research seasons, but they also occurred in the greatest quantities among all sites under analysis, particularly during the period of summer to autumn (Fig. 3).

Seventeen species of fungi were isolated from all sites: 12 mold and 5 yeast-like. The dominating species throughout the entire research period was *Aspergillus fumigatus*. In late spring, it was present at all sites. The greatest variety of isolated species of fungi was observed in the littoral zone of Lake Ruda Woda, and the lowest was in the Lit-1 exposed site of Lake Szeląg Wielki (Table 3).

## Discussion

The acquisition of data concerning the biodiversity and distribution of various groups of bacteria in water bodies forms an important part of each ecological-monitoring process (Drucker and Panasyuk 2006). This information is important because microorganisms respond with changes in both their numbers and community composition to short-term disruptions of ecosystem balance as well as to a long-term supply of allochtonic substances. This response also applies to the environment polluted with toxic substances, such as POPs (Lew et al. 2011). In such ecosystems, microorganisms play a particularly important role, showing the ability to accumulate, detoxify, and metabolize compounds that are toxic for the environment. They often use such compounds as a source of carbon (DeLorenzo et al. 2001). In contrast, the presence of selected toxic substances, such as pesticides, can be a factor limiting the occurrence of some bacteria and fungi, which is also an indicator of ecosystem condition.

In this study, observations of seasonal dynamics patterns concerning the abundance of microorganisms showed a similarity for all sites. Spring thaw and surface runoffs, carrying organic matter from the environment, resulted in a slight increase in bacterial count at all sites, with the highest values recorded in summer and autumn. This finding is a characteristic feature of eutrophic lakes (Lew et al. 2011). Favorable thermal–aerobic conditions and an increased amount of quite easily assimilable nutritional substrates affect the processes of intensive microbiological degradation. This in turn drove the quick growth of bacterial populations (Ploug and Grossart 2000; Grossart and Ploug 2001) that we observed in our research. In addition, macrophytes provide a source of increased supply of organic matter in the littoral zone (Kominkova et al. 2000; Wu et al. 2010, Obolewski et al. 2011). The presence of water plants is positively correlated with the amount and the biomass of microorganisms, which also affects their biodiversity (Wu et al. 2010; Ng et al. 2010). It has been reported that on the reed *Phragmites australis* alone, >600 species of fungi can be isolated (Gessner and van Ryckegem 2003; Wu et al. 2010). This species was the main macrophyte component of the littoral zones at the sites we examined (Grzybowski et al. 2005).

The research indicated that despite the presence of DDT and its traces in the water at the Lit-1 exposed site, there was no modifying effect on the bacteria count. However, the presence of this pesticide in water could have been a factor limiting the presence of fungi. A significant decrease in fungal species diversity was observed for this site. DDT-induced changes in the species diversity index for fungi living in soil and in water have already been reported (Boyer and Perry 1973). In our research, *A. fumigatus* was the most often identified fungal species. It is a saprophyte fungus that plays a key environmental role in carbon and nitrogen circulation (Haines 1995; Latge 1999). In natural ecological niches, the survival and growth of this species is determined by organic remains, large amounts of which are observed in each littoral type in the form of dead macrophyte material and deposited allochtonic waste, such as leaves, wood, twigs, dead animals, insects, and seeds (Czeczuga et al. 2002, 2005; Nechwatal et al. 2008; Wu et al. 2010). However, at the site where DDT was observed in water, *A. fumigatus* was the only isolated representative of fungal taxa. Studies have shown the ability of fungi to biodegrade pesticides in the water environment. Mineralization is performed by ligninolytic fungi, particularly white rot fungi (Aislabie et al. 1997; Jauregui et al. 2003; Thomas and Gohil 2010). In contrast, no reports concerning any metabolic predisposition of *A. fumigatus* for DDT decomposition in water are available; therefore, its presence at this site could be attributed to an outstanding ability to adapt to each environment resulting from its uncomplicated spreading mechanism and, consequently, fast colonization of the ecosystem (Latge 1999; Wu et al. 2010). The seasonal differences in the size and structure of fungal community in the control lake may be caused by the inflow of biogenes into the reservoir. After the spring melt and the inflow of substances that form the nutritional basis, an increase in number and diversification of fungal species is observed in eutrophic lakes (Dynowska et al. 2001; Luo et al. 2004). Our studies have confirmed this relationship. The highest number and most diverse profile of species in Lake Ruda Woda were reported in summer and observed until autumn.

In this study, we examined whether any long-lasting effect of the pesticide tomb was identifiable in terms of the diversity of microorganisms of the *Eubacteria* domain. With this aim in view, we analyzed a pattern of seasonal changes to bacterioplankton using FISH (Pernthaler et al. 2001a, b; Fazi et al. 2005; Souza et al. 2007; Gerbersdorf et al. 2009; Lew et al. 2011). By this method, including use of the HGC 69a probe, we observed the dynamics of Actinobacteria. This was the group that, in our research, made up the greatest share of microorganisms in all seasons, with particularly high abundance in the spring period. Actinobacteria are commonly found in soil, where they decompose organic compounds and play an important role in mineralization. However, they also inhabit water systems (particularly in places with an inflow of allochtonic organic matter), and their abundance in an ecosystem marking a transitory zone between those two environments is to be expected (Rheims et al. 1999). As research has shown, this group is one of the most numerous in lakes, sometimes accounting for approximately half of all microorganisms identified in inland waters (Burket et al. 2003; Van der Gucht et al. 2005; Allgaier and Grossart (2006). Our observations confirm these reports: In the control lake, Lake Ruda Woda, the share of this group reached 40 % in summer in relation to TBC-DAPI. Slightly lower values were observed in Lake Szeląg Wielki, particularly at the site where the presence of DDT was detected. These bacteria, particularly *Streptomyces* and *Micromonospora*, can grow in the presence of chloro-organic pesticides (Benimeli et al. 2003, 2006, 2007; Cuozzo et al. 2009). Experimental studies have shown that in an environment that has not been previously polluted with pesticides, bacteria and actinomycetes are DDT sensitive; however, in a polluted environment many of these microorganisms are not affected or even stimulated by this pesticide. Some investigators have suggested that some Actinobacteria show adaptive abilities to survive in an environment polluted with DDT (Benimeli et al. 2003), which is also supported by our findings from the Lit-1 exposed site.

In our research, we also paid particular attention to two other groups of microorganisms predisposed to mineralize compounds that are difficult to decompose in the environment: *Alcaligenes* spp. of the Beta subclass of Proteobacteria and *Pseudomonas* spp. of the Gamma-subclass of Proteobacteria. Nadeau et al. (1994, 1998) and Parsons (1995) observed that the biphenyl-degrading enzymes of *Alcaligenes* spp. could attack, in a broad scope, aromatic compounds and prove useful in DDT degradation. We also observed a large representation of this group of bacteria in the bacterioplankton, with particular focus on the site where DDT was detected. Here, the participation of *Alcaligenes* within Betaproteobacteria was slightly greater than at other sites and did not decrease below 15 % of all Betaproteobacteria. The presence of chloro-organic pesticides in the water of this littoral zone was confirmed by the low sensitivity of this bacterial group to pollutants reaching the environment (Gonzales et al. 1998). However, Beta-subclass of Proteobacteria is a dominating fraction of freshwater microorganisms, particularly in the pelagial zones (Pernthaler et al. 1998; Klammer et al. 2002; Pesce et al. 2006; Lew et al. 2011).

Gammaproteobacteria do not make a numerous group in the lake bacterioplankton. Most often, they form a community of microorganisms living in the river biofilm or detritus (Kirchman 2002). The share of this group of proteobacteria in the community of littoral microorganisms at a level of approximately 6 % to 11 % in relation to all identified microorganisms brings these data closer to the results obtained in analyses of marine coastal waters (Cottrell and Kirchman 2000a, 2000b). Regarding the Gamma subclass of Proteobacteria, emphasis was placed on *Pseudomonas* spp. As shown by previous research, this group of microorganisms can participate in biodegradation of herbicides and chloro-organic pesticides (Nawab et al. 2003; Kamanavelli and Ninnekar 2000, 2004; Koneva 2004; Li-feng et al. 2007). In the natural environment, under aerobic conditions, *Pseudomonas* spp. can degrade chloro-organic compounds, including DDT, to 4-chlorobenzoic acid. Previous studies have indicated that it is from ecosystems polluted with insecticides that these microorganisms can be successfully isolated on culture media enriched with biphenyl (Kamanavelli and Ninnekar 2004). In our research, the share of *Pseudomonas* spp. was also observed in the community of plankton microorganisms of littoral zones. Nevertheless, it was not proven that the presence of DDT observed at the Lit-1 exposed site could have in any way affected their share in the community of microorganisms. A clear increase in summer, also in the form of compounds that are difficult to decompose but that *Pseudomonas* bacteria specialize in metabolizing, confirmed the presence only of organic matter in water during that period.

Cytophaga-Flavobacterium phylotypes are chemo-organotrophs whose basic function in water ecosystems is dissolved organic matter uptake and degradation. They decompose biopolymers, such as cellulose and chitin—*i.e.*, the high molecular–mass fraction—that are a part of the dissolved organic matter (Kirchman 2002). The high molecular mass of the dissolved organic matter fraction is most intensively released from aggregates and detritus during spring water circulation and after the matter reaches the reservoir with surface runoff. The second period in which the lakes are enriched with this type of matter is autumn decomposition of macrolytes (Manz et al. 1996; Kirchman 2002). The Cytophaga-Flavobacterium group in our studies was characterized by a larger share throughout the entire research period in spring and by a slightly lower, but also recorded, growth of this group in autumn. No effect of the pesticide presence was observed on the seasonal dynamics of these microorganisms.

The presence of pesticides in water inevitably affects inhabitants of these ecosystems. Unfortunately, the harmful influence of pesticides on microbial species subsequently affects organisms at greater trophic levels (DeLorenzo et al. 2001; Friberg-Jansen et al. 2003). A littoral zone, acting as a buffer protecting lakes against external pollution, is where many substances reaching the reservoir from the environment collect. Microorganisms inhabiting this area respond to the presence of pesticides washed out from the drainage area. They participate in the decomposition of harmful substances, thus contributing to detoxification of the environment they inhabit. Long-term exposure to the presence of pesticides can stimulate adaptive abilities in many microorganisms and contribute to participation of those substances in metabolic transformations. In addition, bacteria and fungi, because of quick changes in quality and quantity composition of the community, provide a convenient indicator of the status of the ecosystem they inhabit and can be used for its monitoring. Our environmental research confirmed those assumptions, which have thus far been verified only through laboratory experiments. The quantity dynamics of bacterioplankton showed here a pattern that is characteristic for eutrophic water reservoirs—the lack of response of the littoral community to the presence of pesticides—and confirms bacterial adaptation to seasonal exposure to DDT. In addition, slight changes in the percentage share of individual large groups of bacteria can indicate changes in the environment and suggest the need to monitor such ecosystems. Nevertheless, they unquestionably indicate a high flexibility of the water environment and adaptive abilities of microorganisms as well as their participation in the struggle for preserving the balance of the ecosystem they inhabit.

Determination of the share of *Alcaligenes* spp. in the community proved more useful than determination of the share of *Pseudomonas* spp. as an indicator of changes within the bacterioplankton caused by the DDT presence in water. Experimental data were confirmed, showing on one hand a high susceptibility of selected fungal species to toxic substances and proving in contrast that a long-lasting inflow of harmful substances results in a decrease in fungal species diversity.
